# Effects of α subunit substitutions on the oxidation of βCys93 and the stability of sickle cell hemoglobin

**DOI:** 10.1080/13510002.2020.1834250

**Published:** 2020-10-15

**Authors:** Wayne Hicks, Fantao Meng, Tigist Kassa, Abdu I. Alayash

**Affiliations:** a Laboratory of Biochemistry and Vascular Biology, Center for Biologics Evaluation and Research, Food and Drug Administration (FDA), Silver Spring, Maryland, USA; b Hemoglobin Oxygen Therapeutics, Souderton, Pennsylvania, USA

**Keywords:** Sickle cell hemoglobin mutants, oxidation, βCys93

## Abstract

The β subunit substitutions, F41Y and K82D, in sickle cell hemoglobin (Hb) (βE6 V) provides significant resistance to oxidative stress by shielding βCys93 from the oxidizing ferryl heme. We evaluated the oxidative resistance of βCys93 to hydrogen peroxide (H_2_O_2_) in α subunit mutations in βE6 V (at both the putative and lateral contact regions) that included (1) αH20Q/βE6 V; (2) αH50Q/βE6 V; (3) αH20Q/H50Q/βE6 V; (4) αH20R/βE6 V; and (5) αH20R/H50Q/βE6 V. Estimation by mass spectrometry of irreversible oxidation of βCys93 to cysteic acid (CA) was unchanged or moderately increased in the single mutants harboring a H20Q or H50Q substitution when compared to control (βE6 V). The introduction of Arg (R) singularly or in combination with Q enhanced the pseudoperoxidative cycle by slightly decreasing the ferryl in favor of ferrous and ferric species after treatment with H_2_O_2_. Higher rates for heme loss from the ferric forms of the Q species to the receptor high affinity recombinant apomyglobin were observed in contrast to the R mutants and control. Because of their improved solubility, a combination of Q and R substitutions together with mutations carrying redox active variants (F41Y/K82D) may provide dual antioxidant and antisickling targets in the design of gene therapy-based candidates.

## Introduction

Sickle cell disease (SCD) has been described as a ‘molecular disease’ due to the substitution of a single amino acid valine (Val) for glutamic acid (Glu) at position 6 of the β globin chains of hemoglobin S (HbS) [1]. The replacement of a negatively charged Glu by a non-charged amino acid Val, causes inter-β subunit hydrophobic packing between positionally equivalent amino acids. This hydrophobic effect leads to polymerization of Hb molecules during deoxygenation causing the formation of long Hb fibers, RBC deformation (sickling), membrane instability, and cellular rupture (hemolysis). This leads to a markedly shortened RBC’s life span, vaso-occlusive events and organ damage [2].

The continuous oxidation of the heme iron in Hb (autoxidation) and impaired antioxidant capacity promote a pro-oxidative environment in sickle RBCs, thus compromising the overall redox balance in these cells and impairing their metabolic processes [3]. For instance, ferrous HbS (Fe^2+^) autoxidizes to the non-functional ferric (Fe^3+^) (metHb) form faster than normal Hb (HbA), despite the presence of reducing enzymes, causing a considerable metHb accumulation inside the RBCs [3]. Both ferrous and ferric Hb in the presence of excess reactive oxygen species (ROS), mainly H_2_O_2_ generated within RBCs, or from the plasma compartment, are additionally oxidized to the ferryl Hb (Fe^4+^) through a pseudoperoxidative cycle [4].

Ferryl Hb in sickle RBCs and its associated protein radicals (·Fe^4+^) are highly reactive and toxic species that can be internally damaging to the protein itself and other biological molecules. Because of the ferryl HbS redox reactivity, a higher level of intra-β oxidation occurs at key amino acids on the protein such as βCys93 and a handful of other ‘hotspot’ residues [4]. The irreversible oxidation of βCys93 and other ‘hotspot’ residues destabilizes the Hb molecule resulting in high protein turnover and heme loss [5]. Heme is considered an important damage associated molecular pattern (DAMP) molecule and triggers an inflammatory response and vaso-occlusion through the activation of the toll-like receptor 4 (TLR4) in SCD murine models [6].

We have recently shown in the Townes sickle cell mouse model that the transformation of Hb to higher oxidative states (ferryl) in RBC-derived microparticles (MPs) can occur in the circulation of these mice [7]. Moreover, we found that HbS oxidation (coupled with changes in cytosolic antioxidative proteins) resulted in RBC membrane alterations and RBC MP formation in Townes-SS mice. Consequently, ferryl Hb was implicated in RBC membrane alterations as well as cellular and subcellular changes in the vasculature. In addition to βCys93 oxidation, ferryl Hb also induced complex formation with band 3 and other membrane proteins and activation of the Ubiquitin Proteasome Pathway (UPS pathway) [7]. Similar Hb-driven oxidative changes were also reported in blood samples from SCD patients in response to cellular oxidative stress [8].

To control the damaging ferryl heme in SCD, we recently constructed three HbS variants. The first contained a redox-active Tyr (replacing Phe) (F41Y) in the β subunits, a substitution present in human mutant Hb Mequon. Whereas the second contained Asp replacement of Lys (K82D) found in the β cleft of Hb Providence. The third construct contained both β subunit variants [9]. In the presence of H_2_O_2_, βF41Y and βF41Y/K82D enhanced ferryl Hb reduction by providing a pathway for electrons to reduce the heme via the Tyr41 side chain. Whilst Asp82 provided oxidative stability through appropriate local folding of the protein. Mass spectrometric analysis of βCys93 revealed moderate inhibition of thiol oxidation in the HbS single F41Y variant and a dramatic 3- to 8-fold inhibition of cysteic acid formation in the rHbS βK82D/βE6 V and βF41Y/K82D/βE6 V protein variants, respectively. Substitutions of βK82D/βE6 V and βF41Y/βE6 V not only added significant resistance to oxidative stress, but also provided an additional anti-sickling property by delaying polymerization of HbS [9].

Numerous natural or recombinant HbS mutants with substitution(s) at the putative contact sites have been constructed for the purpose of investigating the mechanism of inhibition of HbS polymerization, although some of these studies have yielded conflicting results. For instance, the structural, functional, and polymerization properties of five recombinant HbS mutants: αH20R/βE6 V, αH20Q/βE6 V, αH50Q/βE6 V, αH20R/αH50Q/βE6 V, and αH20Q/αH50Q/βE6 V were recently reported to offer minimal amounts of stability in HbS polymers [10]. It was shown that the putative axial interaction between αHis-20 →Gln, has minimal contribution in stabilizing the HbS polymers. However, an earlier report showed that this mutation increased the polymerization delay time of HbS [11]. Nevertheless, it has been consistently demonstrated that the solubility of HbS can be markedly improved by replacing the αHis-50 with a Gln. Specifically, αH50Q/βE6 V is nearly 4 times more soluble than HbS [10]. Furthermore, the αH50Q/βE6 V mutant also improves the solubility of (βE6 V/αH20R) and βE6 V/αH20Q, and to date the αH50Q/βE6 V mutant is the most soluble HbS variant reported [10].

In this investigation, we subjected α subunit mutants to oxidative stress to test the hypothesis that amino acid variants in the α subunit can provide resistance to oxidative stress caused by H_2_O_2_. Accordingly, we determined the levels of ferryl Hb formation, its stability and autoreduction to ferric Hb. We also calculated the rates of heme loss from the mutants studied to a receptor mutant apomyglobin. The conclusions provided from the study on site-specific mutations in the HbS molecule provide a framework for Hb prototypes that exhibit favorable anti-oxidant and anti-sickling features as a potential target for genome-editing technologies.

## Materials and methods

### Recombinant protein expression and purification

The expression vectors constructed in this study were derived from the pHE230 plasmid [10]. The pHE230 plasmid encodes the *Escherichia coli* methionine aminopeptidase and synthetic human α- and β-globin genes under the control of separate tac promoters. The β-globin gene carries a βGlu-6 to Val substitution for the expression of recombinant Hb S proteins. The pHE230 was used as template in polymerase chain reactions to generate plasmids pHE295 (αH20Q/βE6 V)), pHE2012 (αH20R/βE6 V), pHE296 (αH50Q/ βE6 V), pHE297 (αH20Q/αH50Q/βE6 V), and pHE2022 (αH20R/αH50Q/βE6 V). The mutations on the plasmids were confirmed by DNA sequencing analysis of the entire α- and β-globin cDNAs [10].

### Isoelectric focusing

Isoelectric focusing (IEF) analyses of Hb and variant samples were carried out on precast IEF-agarose gels (Invitrogen®, pH 3–10, IEF gel). The gels were electrofocused using a Novex® power supply at 100 V for 1 h, 200 V for 1 h, and 500 V for 30 min. The IEF gels were fixed in 12% TCA for 30 min.

### Reversed-phase high-performance liquid chromatograph (RP-HPLC)

RP-HPLC was performed with a Zorbax 300 SB C3 column (4.6 × 250 mm) coupled to a Waters HPLC system consisting of a Waters 626 pumps, 2487 dual-wavelength detector, and a 600-s controller installed with Empower 2 (Waters Corp, Millford, MA). 20 μg of Hb in 25 μL of water was loaded onto a C3 column equilibrated with 35% acetonitrile containing 0.1% TFA. Globin chains were eluted with a gradient of 35–50% acetonitrile within 100 min [5] 100 mins to go from 35 to 50%, or for the whole gradient at a flow rate of 1 mL/min. The eluent was monitored at 280 nm for globin chains and at 405 nm for the heme component.

### Spectrophotometry and hemoglobin concentration measurements

Hb concentrations were calculated on a heme basis. The levels of ferrous/oxy-Hb, ferric/metHb, and ferryl Hb were measured based on the absorbances at λ = 541, 560, 576, 630, and 700 nm using recently published extinction coefficients [12]. Spectrophotometric measurements were carried out in a UV-visible diode array spectrophotometer (Agilent HP 8453).

*Spectrophotometric assessment of mutant hemoglobins after oxidation with peroxide-* Reaction mixtures of ferrous Hbs (60 µM) with molar excess H_2_O_2_ (60, 150, and 600 µm) were monitored every 20 s for 30 min in a photodiode array spectrophotometer (HP 8453). After completion of the reaction, 2 units of catalase were added into the reaction mixture (1 mL) and incubated for 1 min to remove excess H_2_O_2_. Concentrations of individual redox forms of Hb were calculated on a heme basis using recently published extinction coefficients [12]. Spectrophotometric measurements were carried out in a UV-visible diode array spectrophotometer (Agilent HP 8453).

### Kinetics of heme loss from ferric mutant hemoglobins

Absorbance changes were monitored after mixing the double-mutant apomyoglobin (apoMb) (heme acceptor) (H64Y/V86F) with samples of met form of each mutant. The apoMb reagent binds heme released from ferric Hb generating a unique spectrum for the ‘green’ holoMb end product as described previously [13]. Absorbance spectra between 350 and 700 nm were recorded every 2 min for 16 h at 37°C using 200 mM potassium phosphate buffer at pH 7.0 to which 600 mM sucrose was added to prevent denaturation of the resultant apoHb. To carry out this experiment under first-order reaction kinetics, excess heme-binding ApoMb was used. The final concentration of ferric Hb in heme equivalents was 2 μM, and the final concentration of H64Y/V86F apoMb was 20 μM in a 1-ml reaction volume. Data collection started immediately after mixing the two solutions; absorbance changes at 410 nm were used for the calculation of the extent of heme transfer [13].

### Mass spectrometric analysis of cysteine 93 oxidation by peroxide

#### Trypsin Digestion

The oxidized samples were precipitated using trichloroacetate, and dried using a vacuum concentrator. The pellet from each sample was suspended in 6.0 µM urea, 20 mM DTT and 50 mM NH_4_HCO_3_, pH 8.0. The solution was then diluted 3x to a final urea concentration of 2M using 50 mM NH_4_HCO_3_, pH 8.0 for 1 hr incubation time at 37°C. Following incubation, the samples were centrifuged for 30 secs followed by alkylation with 10 µM Iodoacetamide before treatment with trypsin at a 1:100 trypsin: protein ratio.

#### Sample Preparation for LCMS analyses

The trypsin digested samples were desalted using µC18 Ziptips® following the standard protocol provided by Millipore. These samples were then lyophilized and reconstituted with 2% of 0.1% trifluoroacetic acid to a final volume of 50 µL and stored at −20°C. For LCMS analysis 20 µL was brought to a final volume of 31 µL (1 pmol/µL). The samples were further diluted to 0.25 pmol/µL prior to LCMS analysis.

#### LCMS analyses

The trypsin digested samples were analyzed using a Waters Xevo QTOF mass spectrometer equipped with a nanospray source, coupled to a Waters NanoAcquity liquid chromatography system. The samples were injected onto an Acquity UPLC Symmetry C18 10 K 2 g V/M trapping column, and eluted onto an Acquity UPLC peptide BEH C18 column 130 A, 1.7, 75 µm × 250 mm. In-line fractionation was done using a two-phase gradient system. Solvent A was 2% acetonitrile (ACN) in 0.1% formic acid and (solvent B)100% ACN was 0.1% formic acid. The mass spectrum acquisition was done in positive-ion survey mode using a data-dependent method. The mass acquisition window was set for 100 −1990m/z in full MS mode. The detector was set to positive ions in continuum mode. An intensity threshold was set for 50 counts /sec with a scan time of 0.9 sec. MS/MS mode was set with a mass window of 50–1990 m/z. Three MSMS scans were collected for each MS peak with charge state peak selection from +1 to +4. The collision energy was ramped according to charge.

#### Data analyses

Each sample was run in triplicate. Mascot was used for the protein database search. Raw files were searched against an IPI database that was modified to contain the sequences of all of the Hb variants that we have analyzed in our lab. This database contained 69,018 proteins for the initial analysis of the HbS mutants, and controls. It was later updated to contain 69,026 proteins. Database searching parameters included variable modifications for carbamidomethyl cysteine, oxidized and deoxidized methionine and cysteine trioxidation were selected. A mass error of 40 ppm for MS, and 0.1 Da for MSMS was used. One missed cleavage site was specified. The Mascot search results for the βE6 V protein of αH20Q/H50Q/βE6 V mutant were resubmitted to an error tolerant search to identify the carbamylated forms of the β E6 V 84–105 (GTFATLSELHCDKLHVDPENFR) peptide. The data files from Mascot were then analyzed using Scaffold v.4.10 for validation of peptide identifications. Two peptide identifications with at least a 95% probability were required for a positive protein identification with a 90% protein probability. The Scaffold results were then analyzed using Scaffold PTM v.3.3 for positive confirmation of post-translational modifications. Ion abundances and spectral counts were also generated using Scaffold PTM.

## Results

### Recombinant proteins

**Figure 1.**
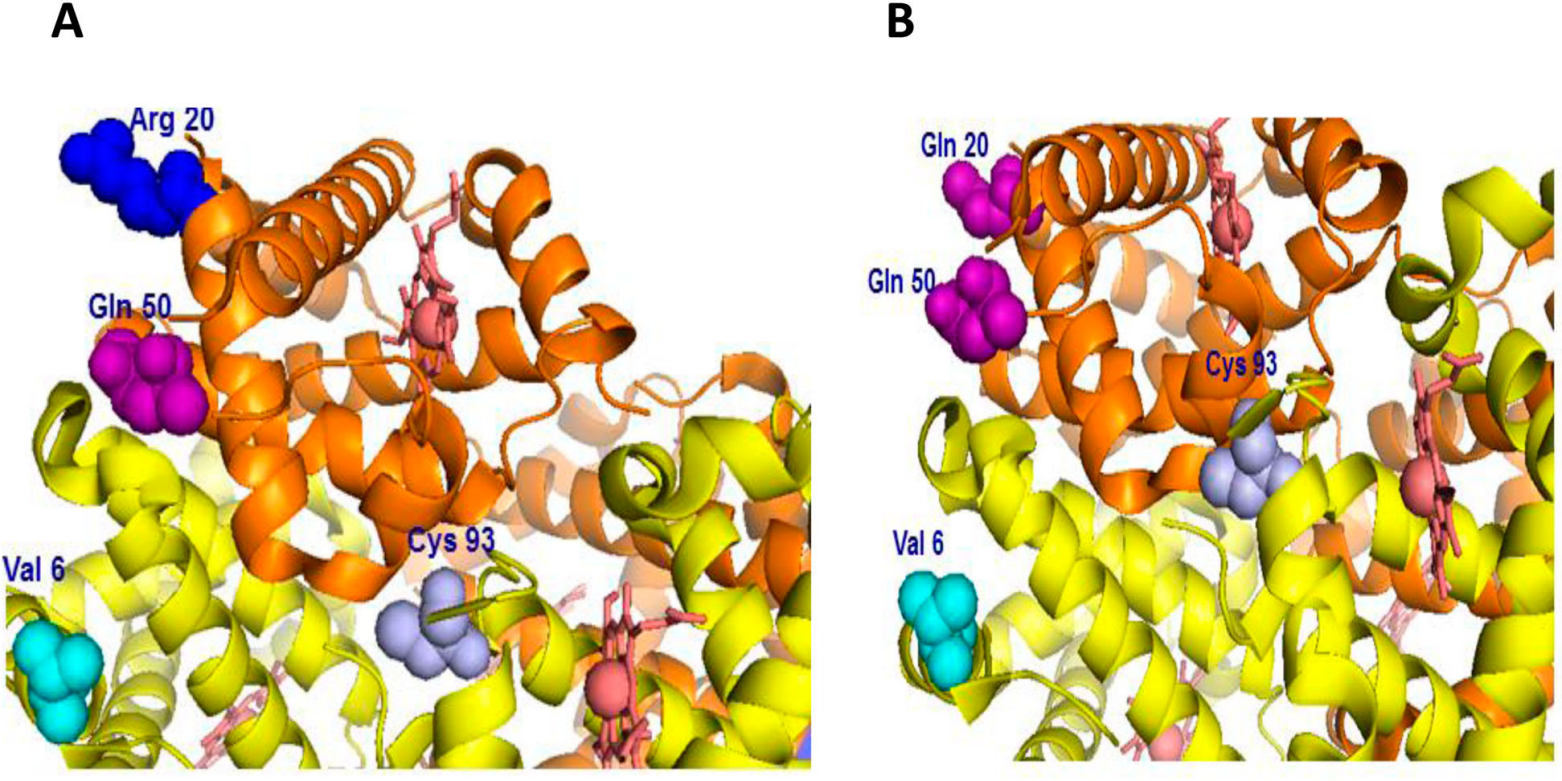
Model structure of human hemoglobin showing sites of amino acids replacements in the α subunits (two separate orientations A and B). Arg20 (blue), Gln50 (purple) and Gln20 (purple) in relation to β subunit sites of mutation Val6 (cyan) and the target of oxidation Cys93 (light blue). Images in both panels were generated from Protein Data Bank code 1XZ2.

A representation of human Hb in which specific amino acid mutations are indicated in relation to key amino acids, e.g. βCys93 (target of ferryl mediated oxidation) and βVal6 (site of original genetic mutation in HbS) in Figure 1(A and B). These novel recombinant proteins were expressed with a single amino acid substitution at the putative axial (αH20R/βE6 V) and (αH20Q/βE6 V), lateral (αH50Q/βE6 V), or combined double amino acid substitutions (αH20R/αH50Q/βE6 V) and (αH20Q/αH50Q/βE6 V) [10].

### Isoelectric focusing and reversed HPLC characteristics of mutants

**Figure 2.**
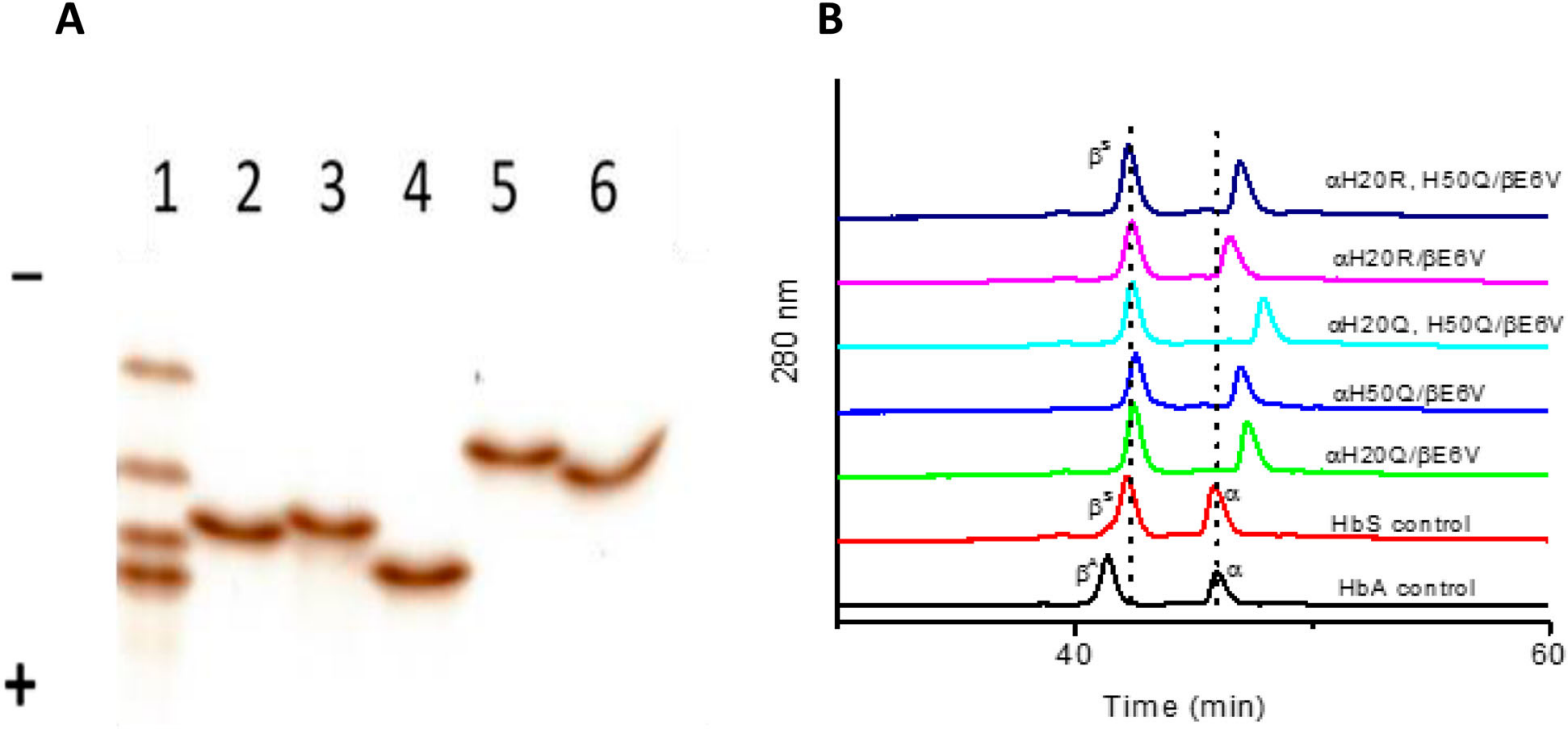
Electrophoretic and HPLC analyses of mutant hemoglobins- A) IEF analyses (n=3) of mutant Hbs. Lane 1, AFSC (from anode up: HbA, HbF, HbS, and HbC); lane 2, αH20Q/βE6 V; lane 3, αH50Q/βE6 V; lane 4, αH20Q/H50Q/βE6 V; lane 5, αH20R/βE6 V; lane 6, αH20R/H50Q/βE6 V. B) RP-HPLC analyses of α and β subunits of mutants and control (HbA and HbS). RP-HPLC analyses (n=3) was performed using a Zorbax 300 SB C3 column (4.6 X 250 mm). Hb (20 µg) in 25 µl water was loaded on a C3 column equilibrated with 35% acetonitrile containing 0.1% TFA. The globin chains were eluted with a 35–50% acetonitrile gradient for 100 min at a flow rate of 1 ml/min, and the elution was monitored at 280 nm.

A representative isoelectric focusing (IEF) result is shown in Figure 2(A) for all the mutant proteins used in this study together with the reference standards (AFSC) (bottom to top, HbA, HbF, HbS, and HbC) in a pH range of (3-10) (lane 1). The pI of HbA is about 6.9, whereas that of HbS is close to 7.1 due to losing one negative charge on the Glu residue. The replacement of a positively charged His with a null charged Gln, decreased the net positive charge on HbS mutants αH20Q/βE6 V and αH50Q/βE6 V, and they migrated toward the anode direction as expected (lane 2 and 3 in Figure 2(A)). Additionally, the presence of two Gln residues in the αH20Q/αH50Q/βE6 V mutant led to further migration towards the anode (Lane 4 in Figure 2(A)).

Since the pKa of Arg is higher than His (12.48 vs 6.04), the replacement of His by Arg increased the net positive charge on the recombinant HbS (αH20R/βE6 V) causing it to migrate closer to the cathode (Lane 5). Additional replacement of a weak positive charge of 50H (αH20R, H50Q/βE6 V) with null charge Gln, led rHbS to migrate back towards the anode (Lane 6 in Figure 2(A)).

Reverse phase HPLC profiles of all HbS constructs are shown in Figure 2(B). As expected, the native control β^A^ eluted at 41 min whereas β^S^ eluted at slightly longer time, 42 min, due to replacement of βGlu6 with Val. Similarly, β^S^ globins in all recombinant and native HbS controls eluted at the same position, ∼42 min. In contrast, the α chains (i.e. sites of mutation) of various mutants investigated eluted at different times. The replacement of His with a more hydrophobic Gln increased the elution time from 46 min of α globin to 47 min in the αH20Q mutant. Similarly, the αH50Q mutant also had an elution time of 47 min as it contains the same mutation but at a different site. Double replacement of His with the more hydrophobic Gln (αH20Q/αH50Q/βE6 V) increased the hydrophobicity of α globin delaying the elution time to 48 min. Under acidic conditions, the hydrophobicity of Arg is slightly higher than that of His [14], accordingly, the replacement of His with Arg (αH20R) slightly increased the elution time from 46 min to 46.3 min. Last, there was a further increase in retention time (about 1 min) when H50Q was added to the αH20R single mutant (αH20R/αH50Q/βE6 V), causing it to have a retention time of 47.3 min.

Collectively, these experiments (IEF and HPLC) show that mutants with a single or double substitution in the α subunit have undergone subtle conformational changes due to changes in their overall net electric charge and hydrophobicity.

### Oxidative reactions of mutant sickle cell hemoglobins with peroxide

**Figure 3.**
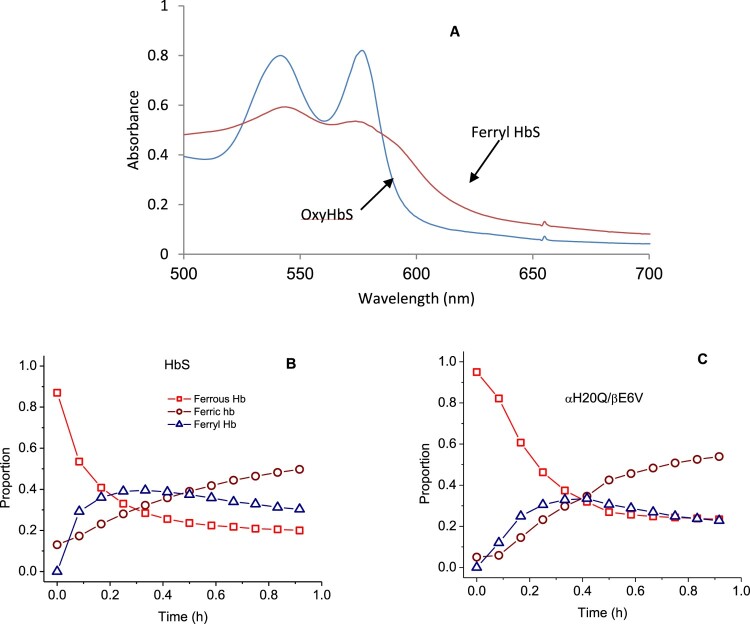
Spectral analysis of peroxide reaction with hemoglobins- (A) Spectral changes during the reaction of 60 µM HbS mixed with 600 µM peroxide in phosphate buffer (50 mM, pH 7.4) at room temperature. (B & C) Time courses of Hb redox states (ferrous, ferric and ferryl) of HbS and the mutant αH20Q/βE6 V. Spectra were collected every 5 min for over a period of 1 hr in a temperature-controlled spectrophotometer.

Reaction of H_2_O_2_ with the oxy/ferrous forms of βE6 V and its corresponding mutants was carried out in a rapid scanning spectrophotometer. We used increasing ratios of H_2_O_2_ over heme (i.e. 1, 2.5, 5 and 10 H_2_O_2_: heme). During a typical experiment (1 heme: 10 H_2_O_2_) (Figure 3(A)), the initial oxidation of ferrous heme can be seen by the gradual loss of α and β bands (577 and 541 nm) and the emergence of a typical ferryl intermediate which absorbs at 545 and 580 nm, and a flattened response region between 600 and 700 nm. The ferryl quickly reverts to metHb with absorbances at 519, 550 and 630 nm completing a typical pseudoperoxidase cycle [5].

Oxidation experiments involving all mutants were carried out under the same reaction conditions. Fractional changes of oxy, met and ferryl for HbA and its SS mutants were obtained for all samples at different ratios of heme: H_2_O_2_. A typical distribution with time for the ferrous, ferric and ferryl species at (1 heme: 1H_2_O_2_)) for βE6 V and αH20Q/βE6 V are shown in Figure 3(B and C) respectively.

**Figure 4.**
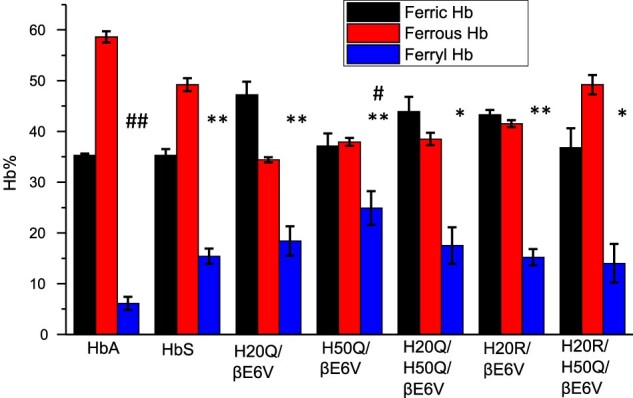
Fractional changes in the redox states of sickle cell hemoglobin mutants- Normalized time courses for the reaction of Hb (60 µM) during the 60 min oxidation with peroxide (60Hb/60 H_2_O_2_) in phosphate buffer (50 mM, pH 7.4) at 25°C. Proportions of each redox state of (ferrous, ferric and ferryl) during the 30-minute time period in which the oxidation reaction was monitored. * indicates *P* < 0.05 vs. ferrylHbA, ** indicates *P* < 0.001 vs. ferrylHbA. Levels of ferrylHb in all mutant Hbs were significantly higher than in the HbA solutions; ^#^ indicates *P*< 0.05 vs. ferrylHbS, ^##^ indicates *P*<0.001 vs. ferrylHbS. For comparison with HbS solutions, H50Q is the only mutant that showed the highest levels of ferrylHb. Statistical calculations were made using Student’s t-test, two-tailed, where *P*<0.05 was considered significant between groups.

A comparison of the relative integrated areas under the ferryl curves (derived from typical time courses for all mutant as shown in Figure 3(B and C) is provided in Figure 4). This represents a quantitative comparison of the ‘dose’ or time persistence of the ferryl species during the oxidation of the ferrous heme of all mutants. Estimates of the amount of ferryl species generated in HbS versus HbA is clearly different and in keeping with our reported model [5]. There is a slight reduction in the amount of ferryl in some mutants. The relationship between oxygen binding capacity (oxy/deoxy) and ferryl levels during oxidation can be explored further since these mutants purposefully had their P_50_ values (oxygen affinity of Hb when half saturated) altered [10]. Generally, Gln (Q) mutations at either residue 20 or 50 showed slight increases in the ferryl species over native HbS controls. However, the double Q mutants retained similar levels of the ferryl species as native HbS and the single Q mutation did not reach statistical significance (see legend for Figure 4). Notably, combining Arg (R) at the 20 position with Gln at residue 50 provided a slightly higher recovery in the ferrous state and lowest ferryl accumulation among all mutant solutions.

### Heme loss from the HbS mutants

**Figure 5.**
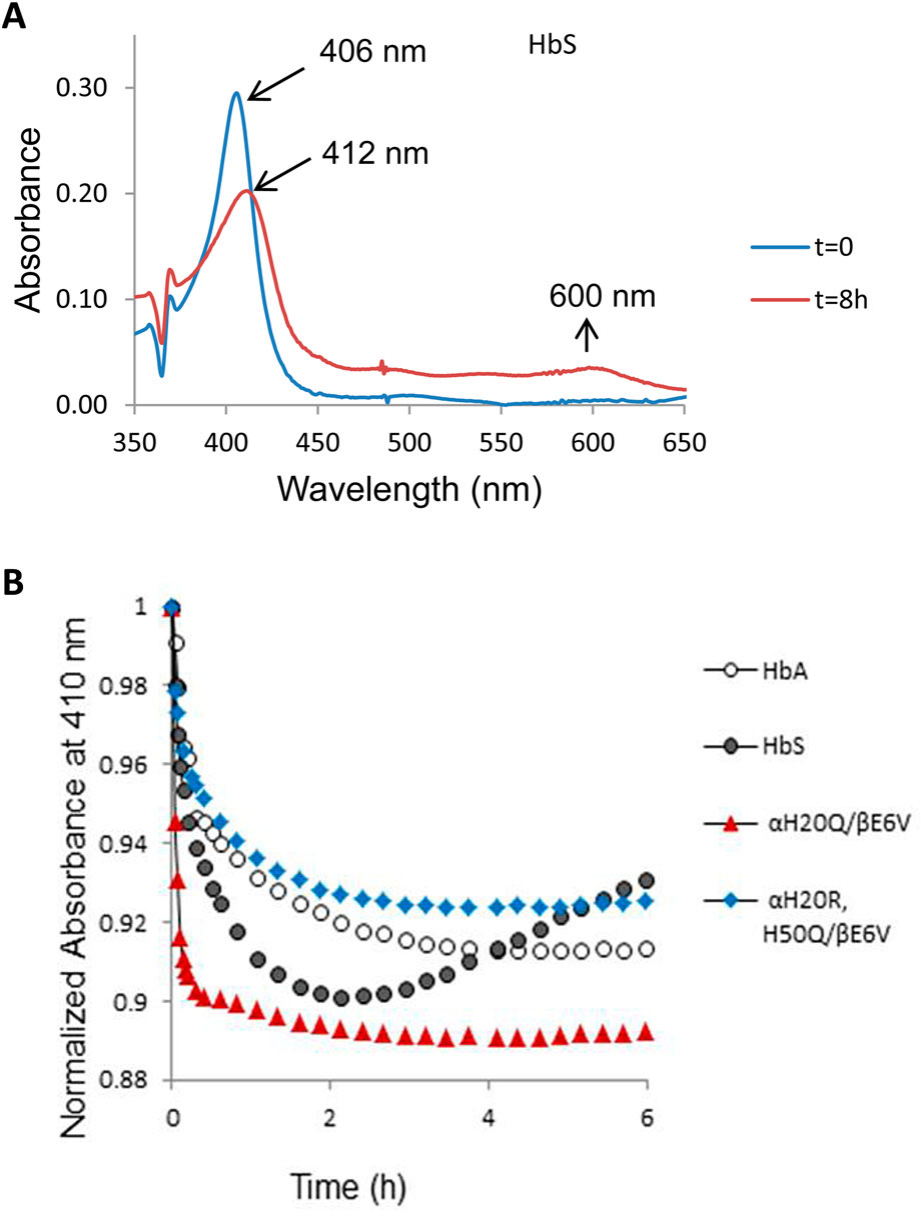
Kinetics of heme dissociation from HbS and its mutants. (A) Absorbance spectra of the ferric (met) form of HbS (blue) and holoMb (H64Y/V86F) (red) during the course of heme transfer. (B) Absorbance changes at 410 nm are plotted as a function of time. Time courses for HbA, HbS and HbS mutants (αH20Q/βE6 V and αH20R/H50Q/βE6 V) were fitted to a double exponential expression. Estimated rates of heme transfer for all mutants and controls are listed in Table 1.

Oxidative pathways described above ultimately lead to unfolding of the protein and loss of heme. To assess the rate of heme release from these mutants, we measured the absorbance changes when a heme acceptor, the double-mutant (H64Y/V86F) apomyoglobin (ApoMb), as it binds the heme released from the ferric forms of HbS and HbS mutants (Figure 5(A)) [13]. Time courses for heme loss measuring the decrease in λ 410 nm as heme transferred to the H64Y/V68F apoMb reagent is shown in Figure 5(B). As shown in earlier reports, the time courses in this study were biphasic with fast components representing heme loss from β-subunits of proteins derived from time courses of absorbance verses time. Table 1 lists the heme loss rates generated for all mutants. The highest heme loss rates were recorded for the single and double mutation where Q is either at the 20 or 50 positions. However, including Arg singularly or in combination with Q lowered the rates of heme loss by almost half (for statistical analyses see Table 1).

**Table 1.** Estimated rates of heme transfer to H64Y/V86F apomyglobin from α subunit mutants.

Rate/ h^−1^	HbA	HbS	H20Q/βE6V	H50Q/βE6V	αH20Q/H50Q/βE6V	αH20R/βE6V	αH20R/H50Q /βE6V
k1 *p* value (vs HbS)	8.37 ± 0.63 0.008	0.46 ± 1.9 -	36.8 ± 0.91 0.0004	26.53 ± 0.67 0.014	36.22 ± 0.53 0.004	6.83 ± 0.63 0.069	23.75 ± 1.1 0.114
k2 *p* value (vs HbS)	0.72 ± 0.21 0.206	1.69 ± 0.82 -	1.04 ± 0.11 0.363	0.93 ± 0.22 0.304	1.01 ± 0.27 0.355	1.41 ± 0.44 0.709	1.14 ± 0.91 0.553

Biphasic rates for the transfer of heme from ferric forms of α subunit mutants to H64Y/V86F apomyglobin compared to Hb A and HbS controls. Statistical tests (*P* values) are indicated underneath each rate

### Mass spectrometric analysis of oxidation of βCysteine 93

**Figure 6.**
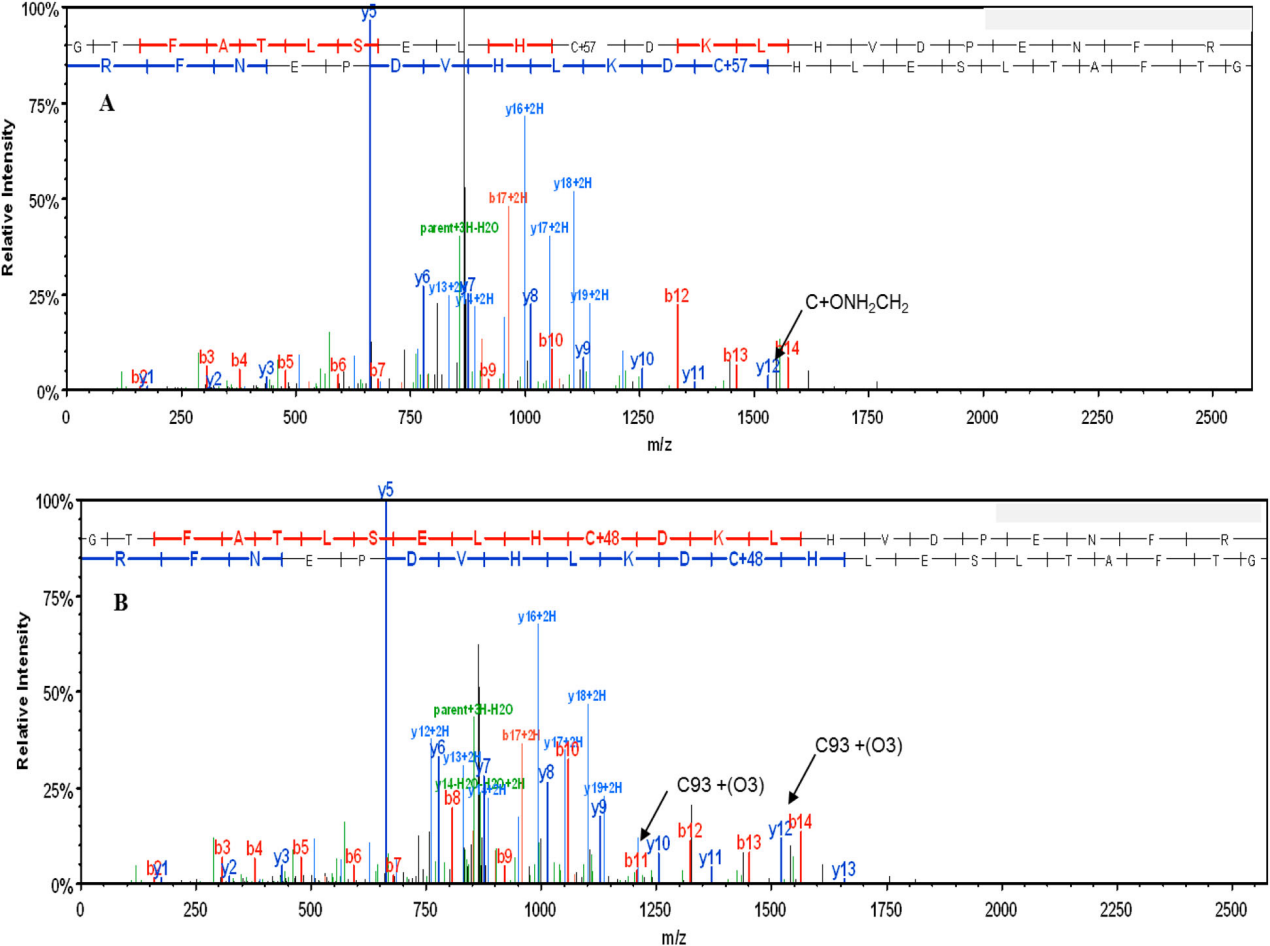
Mass spectrum of the βE6 V tryptic peptide (amino acids 84–105 (GTFATLSELHCDKLHVDPENFR)-Panel A shows the unoxidized peptide that was alkylated prior to proteolysis with trypsin. This resulted in the addition of a carbamidomethyl group to C93. The corresponding ion is indicated by the arrow. Panel B shows spectra of the same peptide following oxidation of C93 to C(O3) by H_2_O_2_ prior to alkylation and proteolysis. The C93(O3) ions from the peptide ladder are indicated by the two arrows.

Figure 6 shows a mass spectrum for a+3 charged tryptic peptide of Cys containing βE6 V (84–105) (GTFATLSELHCDKLHVDPENFR). Panel A shows the mass spectrum from the HbS negative control sample that was not oxidized with H_2_O_2_. The ion corresponding to C93 (βCys93) shows a mass increase of 57 amu due to modification by a carbamidomethyl group during the denaturation and alkylation. Panel B shows the +3 charged tryptic peptide HbS βE6 V (84–105) (GTFATLSELHCDKLHVDPENFR) peptide following oxidation with H_2_O_2_. Oxidation of C93 to C(O_3_) 93 is indicated at the top of the frame where C+48 ion is indicated.

**Figure 7.**
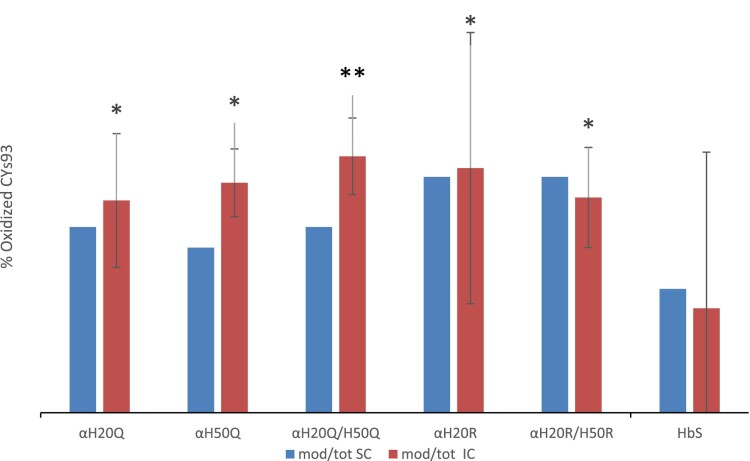
Total fraction of cysteine oxidation in mutant hemoglobin based on mass spectral counting and ion intensity for the 1:5 Heme:H_2_O_2_ sample-Bar graphs (A and B) The Y axis shows the ratio of βE6 V peptides that contain theC93 peptides that were oxidized to cysteic acid as a percentage of the total C93 containing peptides. Each column is an average of three separate LCMS analytical runs. The error bars show the % C.V for each of the individual mutants. SC stands for spectral counting, and IC stands for ion current. *P* values for IC were calculated for the C93 O3 ratios using a two-tailed students t-test. A *P* value <0.05 was considered significant. The * symbol indicates a *P* value > 0.5 and ** symbolizes a *P* value < 0.05.

We used both spectral counting (SC) and ion current (IC) for relative quantification at single peptide level [15]. We carried out three LC/MS analyses for each mutant, together with HbS, and HbA at each ratio of heme:H_2_O_2_ (e.g. 1:1, 1:2.5, and 1:5). HbS and HbA were used as positive and negative controls respectively (HbA data is not shown as it was only used to verify lack of oxidation at C93 in normal Hb). To determine the amount of oxidized βCys93, we calculated the ratio of HbS βE6 V (84–105) peptides that were oxidized to cysteic acid relative to peptides that were not oxidized and were alkylated by carbamidomethyl groups. We included only peptides that were fully tryptic (e.g. peptides with a K, or R at each terminus) and had no more than one missed cleavage. Peptides that were identified using Mascot were validated using Scaffold 3.3 and were validated using Scaffold PTM. Spectral count data and ion current were reported from Scaffold PTM v.3.3. Oxidation ratios for each individual analytical run were calculated and an average ratio was calculated for each group. The results of our comparison indicated that the HbS mutant series oxidized at rates comparable to or greater than that seen for HbS (Figure 7).

The calculated C93 oxidation levels measured by SC and IC abundance at higher H_2_O_2_ levels (1:2.5 H_2_O_2_: heme and 1:5 H_2_O_2_: heme) were in close agreement (see Figure 7 for the 1:2.5 H_2_O_2_: heme). Quantitation of cysteine oxidation by spectral counting and ion counts at the lowest level (1:1) however, showed little or no change due to lower abundance in the Cys oxidation. Ion current quantitation indicated that C93 trioxidation occurred in less than 10% of βE6 V 84–105 peptides with a % C.V. below 10%. The only exception was the α H20Q mutant which had the highest C93 trioxidation level at 11.7%, however this measurement was also associated with the highest error. The disparity between the SC and the IC is likely due to the small number of trioxidized β E6 V (84–105) peptides that were detected [16]. We conclude that there was no difference in oxidation levels for the HbS sample at 1:2.5 and 1:5 heme:H_2_O_2_. Both samples indicate trioxidation of the βE6 V (84–105) peptide of 34.3% and 35.4% respectively. The mutants oxidized at a similar rate to HbS at 1:2.5 heme:H_2_O_2_. However, the αH20R/βE6 V mutant appears to show a lower level of oxidation than the other samples tested at 1:2.5 heme: H_2_O_2_.

The results from the oxidation experiments at 1:5 H_2_O_2_ indicate that the HbS α-globin mutant series continues to oxidize of up to ∼80% at the 1:5 H_2_O_2_: Hb ratio. In contrast, the native HbS protein had approximately half the level of βCys93 oxidation, suggesting that the α subunit mutants evaluated may not have favorable oxidative characteristics.

## Discussion

In the presence of oxidants such as H_2_O_2,_ HbS becomes more susceptible to oxidative changes including post-translational modifications of key amino acids. This is due in large part to HbS’s unique oxidative side reactions that result in more persistent oxidizing intermediates such as the ferryl Hb [5]. These Hb-dependent oxidation reactions were shown to occur in blood from sickle cell mice model [7], and more recently in samples from SCD patients [8]. Pseudoperoxidative transformation of Hb to a higher oxidative state, ferryl Hb, fuels oxidative stress within RBCs leading to increased levels of Hb-laden microparticles (MPs) in both SCD mice and in human samples [8]. Because of ferryl HbS redox reactivity, a higher level of intra-β oxidation occurs at target key amino acids on the protein specifically βCys93.

Molecular therapeutic interventions against SCD are currently focused on the use of recombinant technology/gene therapy in which permanent delivery of a corrective or an anti-sickling gene cassette into long-term, repopulating autologous hematopoietic stem cells. This could potentially produce permanently corrected RBCs in patients. A LentiGlobin BB305 vector, used by one manufacturer, Bluebird Bio encodes adult Hb (HbA) with a Thr to Gln (T87Q) amino acid substitution based on early site-directed mutagenesis experiments [17, 18]. HbA/T87Q demonstrated oxygen binding properties similar to wildtype HbA and antisickling activity similar to HbF. Although the inhibitory effects of a mixture of β/α (HbA) or γ (HbF) with HbS within RBCs appears to reduce its polymerization due to the ‘dilution’ effects, these mutations afford no antioxidative protection mechanisms.

Methods controlling Hb-mediated oxidative pathways in SCD have focused in recent years on the use of small molecule drugs or site-directed mutagenesis which specifically targets the βCys93 ‘hot spot’. This residue is readily and irreversibly oxidized in the presence of H_2_O_2_ to cysteic acid resulting in the unfolding and destabilization of the protein [4, 9]. The reactivity for the sulfur-containing amino acids, βCys is consistently more exposed to the surface according to Accessible Surface Area Calculations (ASA) [4] and therefore is more amenable to reaction with H_2_O_2_. We have recently reported that the mass spectral intensity of peptides containing oxidized and non-oxidized Cys can be reproducibly measured using high resolution mass spectrometry and therefore the oxidation status of this residue can be used to reflect the oxidative stress induced during disease pathophysiology, and ultimately functioning as a useful biomarker of oxidative stress [19]. The quantification of this residue has been used by our group and others to assess the degree of oxidative stability of Hb [20–22].

Ho and co-workers have examined mutations in α chains of HbS, which improved solubility and dramatically increased the delay times before the onset of the polymerization process, theoretically allowing more RBCs to escape capillaries of peripheral tissues [10]. Combination, of His → Gln mutations in α subunits at the axial (position 20) and lateral (position 50) contacts in the HbS fiber caused a major 3-fold increase in solubility and dramatically increased polymerization delay times.

In this study, we focused on the reactivity of these recombinant HbS with H_2_O_2_. These reactions, even at stoichiometric levels, occur much more quickly than simple autoxidation and represent potentially significant pathways for Hb degradation and oxidative stress in the vasculature [23]. For the single mutants (H20Q and H50Q) there was no reduction in the levels of ferryl Hb, rather the mutants with Q at the 50th position showed almost double the amount of ferryl over HbS solutions. These solutions retained approximately the same levels of ferric heme but higher oxyforms in the H20Q mutant. The pseudoperoxidative activity with double mutants containing R/Q seemed to result in ferryl heme reduction when compared to the control HbS. Moreover, the presence of R residues suppressed heme loss from the protein, even in the presence of the Gln residues. The modest effect of these α substitutions on the resistance of the HbS variants to oxidation and radical formation are in line with data captured by our mass spectrometric experiments for the stoichiometric estimates of the levels of βCys93 oxidation.

Oxidation of βCys93 to Cysteic (CA) is known to perturb the extensive network of hydrogen bonding and salt bridges at the interface between the β2 FG corner and α1 C-helix, where αTyr-42 is located. We have previously introduced Tyr-41 in the β subunits almost 10.1 Å from the heme iron, which facilitated rapid electron transfer. The substitution of the native βLys-82 for Asp-82, is located ∼18.3 Å away from the β -heme and is not known to be engaged in electron transfer processes. However, the higher oxidative stability caused by the K82D mutation has been attributed to cause changes in reactivity of the βCys93 side chain [9], which may be due to either indirect electrostatic effects or alterations in the local dynamics of the structure.

The α subunits mutations included in the study, however, are located on the protein surface and are at a distance further away from the heme pocket and target amino acid, βCys93 in the Hb tetramer. Both His20 and His 50 are 44.8 and 42.3 Å away from βCys93 respectively whereas the Arg residues both in 20/50 mutants are located approximately 47 and 44 Å away from βCys93. The rates of autoxidation for these mutants were also reported to be similar to that of HbS, but their oxidative side reactions are more profound than that of HbS [10]. If the effects were additive, it would be interesting to consider the favorable properties of an HbS variant with αH20Q/H50Q or αH50Q/H20R and βF41Y/K82D substitutions that we reported earlier [9]. Such an HbS is likely to be as soluble and resistant to oxidation and should therefore provide targeted gene therapeutic modalities.
